# Influence of physical exercise on negative emotions in college students: chain mediating role of sleep quality and self-rated health

**DOI:** 10.3389/fpubh.2024.1402801

**Published:** 2024-05-02

**Authors:** Fan-zheng Mu, Jun Liu, Hu Lou, Wei-dong Zhu, Zhen-cheng Wang, Bo Li

**Affiliations:** ^1^Institute of Sports Science, Nantong University, Nantong, China; ^2^School of Basic Medicine, Nanjing Medical University, Nanjing, China

**Keywords:** physical exercise, self-rated health, sleep quality, chain mediation model, college student

## Abstract

**Background:**

Negative emotions in college students are a significant factor affecting mental health, with suicide behaviors caused by negative emotions showing an annual increasing trend. Existing studies suggest that physical exercise is essential to alleviate negative feelings, yet the intrinsic mechanisms by which it affects negative emotions have not been fully revealed.

**Objective:**

Negative emotions in college students represent a significant issue affecting mental health. This study investigates the relationship between physical exercise and negative emotions among college students, incorporating sleep quality and self-rated health (SRH) as mediators to analyze the pathway mechanism of how physical exercise affects students’ negative emotions.

**Methods:**

A cross-sectional study design was utilized, employing online questionnaires for investigation. The scales included the Physical Activity Rating Scale-3 (PARS-3), the Depression Anxiety Stress Scales-21 (DASS-21), the Pittsburgh Sleep Quality Index (PSQI), and the 12-Item Short Form Health Survey (SF-12), resulting in the collection of 30,475 valid questionnaires, with a validity rate of 91%. Chain mediation tests and Bootstrap methods were applied for effect analysis.

**Results:**

The proportions of university students engaged in low, medium, and high levels of physical exercise were 77.6, 13.1, and 9.3%, respectively. The proportions of students experiencing “very severe” levels of stress, anxiety, and depression were 4.5, 10.9, and 3.6%, respectively. Physical exercise was significantly positively correlated with self-rated health (*r* = 0.194, *p* < 0.01), significantly negatively correlated with sleep quality (*r* = −0.035, *p* < 0.01), and significantly negatively correlated with stress, anxiety, and depression (*r* = −0.03, *p* < 0.01; *r* = −0.058, *p* < 0.01; *r* = −0.055, *p* < 0.01). Sleep quality was significantly negatively correlated with self-rated health (*r* = −0.242, *p* < 0.01). Mediation effect testing indicated that sleep quality and self-rated health partially mediated the relationship between physical exercise and negative emotions, with total effect, total direct effect, and total indirect effect values of −1.702, −0.426, and − 1.277, respectively.

**Conclusion:**

College students primarily engage in low-intensity physical activity. Sleep quality and self-rated health mediate the impact of physical exercise on students’ negative emotions. A certain level of physical activity can directly affect students’ emotional states and indirectly influence their negative emotions via sleep and self-rated health. Regular engagement in physical activities primarily positively impacts emotional states by enhancing mood stability and overall emotional resilience.

## Introduction

Negative emotions in college students are a vital factor significantly impacting mental health (1). College students are at a crucial transition from students to societal individuals, with factors such as interpersonal relationships, academic pressure, job prospects, and social adaptation making them susceptible to various mental health issues (2). Negative emotions refer to an individual’s adverse attitudinal experience toward objective matters and corresponding behavioral responses, often leading to intense physiological and behavioral reactions, including tension, sadness, fear, guilt, anger, contempt, and disgust (3). Anxiety and depression are two common negative emotions among college students, with numerous studies indicating that stress, arising from an inability to adapt to environmental demands, leads to negative feelings and pessimistic beliefs, with symptoms of anxiety and depression quickly emerging under stress (4). In 2023, the Institute of Psychology, Chinese Academy of Sciences, published the 2022 “Mental Health Blue Book” titled “China National Mental Health Development Report (2021–2022),” revealing that the detection rate of depressive emotions among Chinese college students was 10.6%, and the anxiety risk detection rate was 15.8%, with the 18–24 age group showing a depression risk detection rate of 24.1%, significantly higher than other age groups (5). Consequently, the mental health of college students requires heightened attention. More importantly, identifying effective mental health predictors is crucial for preventing negative emotions.

Prior studies have found that physical exercise is crucial for alleviating negative emotions (6–8). Physical exercise is a crucial and effective method for promoting physical health and can also serve as a green, healthy intervention to prevent aggressive behaviors among college students (9). Dollard’s Frustration-Aggression Theory posits that when individuals encounter frustrations leading to unachieved goals and unsatisfied motivations, they exhibit a series of adverse psychological and behavioral reactions (10). Increasing physical exercise can enhance individuals’ resilience, promote the maintenance of positive emotions, and offer a rational explanation for reducing self-harm and aggressive behaviors among (11). This offers a rational explanation for how sports activities can reduce self-harm and aggressive behaviors among individuals. Scholars have conducted electrophysiological measurements on women performing emotional regulation tasks, where the late positive potential in these measurements indicates that women who frequently engage in physical exercise perform better in controlling negative emotions (12, 13). Further research indicates that as college students increase their exercise levels, their scores for emotional disorders tend to decrease gradually, demonstrating the varied impacts of exercise intensity on emotional health (14). Moderate aerobic exercise has been found to reduce negative emotional responses, particularly in individuals who struggle with emotional regulation (15). The involution of education prevents college students from releasing negative attitudes into positive energy through moderate physical activity in their daily lives. This lack of physical activity can lead to reduced dopamine activity and elevated cortisol levels, resulting in various degrees of negative emotions among college students (16). Synthesizing the above research provides evidence for the preventive role of physical exercise against the emergence of negative emotions in college students. Yet, there remains a lack of robust empirical evidence on the internal mechanisms by which physical exercise can influence negative emotions.

Previous studies suggest that one of the reasons physical activity alleviates unpleasant feelings could be related to the quality of sleep (17, 18). Sleep quality is often associated with a high co-occurrence rate of negative emotions, and sleep disturbances usually become quickly apparent in most case (19). Herman’s circadian rhythm theory suggests the existence of an endogenous biological rhythm system within the human body, regulating the cyclic changes in physiological and behavioral activities within 24 h (20). By combing through the literature on behavioral tendencies and neurological changes, researchers have found that sleep influences mood production and emotion regulation through several potential mechanisms. Lack of sleep and sleep disturbances are identified as common symptoms and risk factors for a variety of mental illnesses, especially closely related to anxiety and mood disorders (21–23). Sleep and emotion regulation share common mechanisms at the neurobiological level. A study indicates that emotional events during the day significantly impact sleep, while the quality of sleep at night indirectly affects individuals’ emotional responses to new events the next day (24–26). The effects of exercise on sleep quality and emotional well-being differed between men and women, and poorer sleep quality was strongly associated with daytime dysfunction in individuals (27, 28). Another research focused on the common issues of insufficient sleep, emotional problems among college students, and their negative attitudes toward participation in physical activities. The intervention group showed potential improvements in sleep and mental health after a 6-week program consisting of three 30-min gaming sessions per week (29).

How does self-rated health further impact college students’ negative emotions? Self-rated health is an individual’s subjective evaluation and cognition of their disease burden and an expectation for their overall health status (30, 31). It can stimulate and guide individuals to actively perceive pain and discomfort that are difficult to observe through external means, thereby reducing the risk of illness and death (32). Extensive research indicates that self-rated health status has a strong predictive power for an individual’s risk of death, and there is significant heterogeneity in the responses to self-rated health among different populations (30). Age and gender significantly influence self-rated health status; as individuals age and accumulate life experiences, their assessment of their health tends to become more pessimistic. Concerns about one’s health status may also affect the likelihood of diseases and accidents occurring (33, 34). To a certain extent, individuals’ expectations of self-rated outcomes govern changes in their behavior. Research shows that mind–body main complaints are essential for developing a framework for rating students’ mental health (35, 36). Psychosomatic complaints are associated with university students’ choices and reflections on the future, as well as the negative emotions they experience when facing real-life challenges (37). Therefore, shaping a positive health perspective among college students is very important. Additionally, the reduction in sleep duration and interpersonal issues among peers can lead to the emergence of anxiety and suicidal thoughts. This evidence supports the association between sleep deprivation and potential mental health issues among the youth and elucidates an intrinsic relationship between individual sleep patterns and self-rated health. Such a relationship aligns with the findings of researchers like Meer (38). Research by Chinese scholar Dong Hanyu and others has also confirmed that higher negative emotions correlate with poorer self-rated health status (39). After interaction analysis, there is an additive interaction between negative emotions and physical exercise (40). Shaping the correct health perspective and enhancing cognitive and thinking abilities regarding health can reduce the negative impact of negative emotions on the organism.

A bidirectional relationship exists between sleep quality and self-rated health, with better self-rated health status associated with improved sleep quality (41, 42). A dynamic relationship exists between subjective sleep quality and the emotional state the following day (43). A longitudinal study indicated that graded assessments of patients based on psychological changes and the severity of mental disorders, using mental health self-rated questionnaires and the Pittsburgh Sleep Quality Index, can help patients find inner balance and good health status. A study utilizing brain imaging found that specific brain regions related to emotions are associated with negative emotions and affect the sleep quality of young people (44). Additionally, research has found that sleep deprivation can directly predict physiological problems in female college students, and the decline in self-rated health due to insufficient sleep can lead to increased stress among this group (45).

In summary, this study is based on the framework of psychological and physiological mechanisms by which physical exercise affects college students’ emotions, as described in the existing literature, attempting to establish the pathway through which physical exercise impacts negative emotions in college students. Based on this, the study proposes Hypothesis *H1: Participation in bodily exercise can effectively regulate negative emotions in college students and improve their mental health status*. To further explore the mechanism by which physical exercise affects negative emotions in college students, based on the principles of sleep medicine and psychometrics, starting from college students’ beliefs in health and overcoming illness and their motivation for taking action, it’s essential to understand the role of positive health behaviors in reducing the risk of mental illness. Thus, the study proposes Hypothesis *H2: Physical exercise regulates negative emotions and enhances mental health by improving sleep quality in college students*. Faced with the challenges posed by anxiety and depression, individuals with higher levels of self-rated health believe they can overcome setbacks and will adopt a series of positive health behaviors to cope with adverse life events and reasonably regulate their emotional changes. From this, Hypothesis *H3 is proposed: Sleep quality and self-rated health play a chained mediating role in preventing negative emotions among college students through physical exercise*.

## Materials and methods

### Procedure and participants

The survey targets students enrolled in general higher education institutions in mainland China, and the list of general higher education institutions refers to the Ministry of Education’s “List of National General Higher Education Institutions (as of September 30, 2021).” Inclusion Criteria: Ordinary university students from first to fourth year with good listening, speaking, reading, and writing skills, possessing the cognitive abilities and intelligence required to understand and complete the questionnaire, and participating in this study is voluntary. Exclusion Criteria: Severe personality disorders, such as individuals with significant physical illnesses, prevent them from completing the questionnaire.

### Sampling methods

The survey subjects were selected using stratified, cluster, and multi-stage sampling methods.

### Determination of sampling locations

To ensure the representativeness of the monitoring subjects, each province and city was allocated three sampling locations. The specific practice was as follows: cities under the jurisdiction of each province or autonomous region were selected as sampling locations. Among them, the provincial capital cities were categorized as “Type 1” sampling locations; the other two sampling locations were determined based on the geographical location of the province or autonomous region, selecting one city with an average level of socio-economic development as “Type 2” and one with relatively poor socio-economic development as “Type 3.” The sampling did not adhere strictly to the above principles in municipalities directly under the central government. Still, it was primarily random cluster sampling, with consideration given to the number of sampling locations.

### Determination of sampling units

When selecting sampling units, three primary considerations were taken into account: first, the higher education institutions should be formally established and recorded by the Ministry of Education, including higher vocational colleges; second, units that meet the sampling requirements (such as age, number of participants, grade distribution, etc.); third, units with a specific person responsible for distributing questionnaires who are willing to participate in the monitoring over the long term.

### Grouping and sample size

Participants were divided into two groups based on gender and then into eight categories by grade, with a minimum of 45 participants in each category (e.g., first-year male students). The total sample size for each province (or municipality directly under the central government) was 1,080 participants, with an expected total of 33,480 participants nationwide (excluding Hong Kong, Macao, and Taiwan). In September 2022, the Questionnaire Star software was used for an electronic survey based on administrative classes, yielding 33,369 completed questionnaires. The number of valid questionnaires was 30,475.

### Survey quality control

First, standardization of research protocols and survey implementation, with specific training for investigators before the official survey, creation of standardized introductions, proficiency with questionnaire content, and cautionary notes for filling out the questionnaire. Investigators include student counselors or teachers. The second is establishing data cleaning rules to ensure the external validity of the analysis data. In data preprocessing, entries with logical errors, omissions, inaccuracies, or undistinguishable responses are retested or excluded to ensure data authenticity and validity. The third is conducting tests for common method bias before applying data analysis. During the administration to college students, the principal investigator emphasizes the anonymity and confidentiality of the questionnaire, explaining that the data is solely for scientific research to control for sources of standard method bias as much as possible. Harman’s single-factor test method is also used to test common method bias. The result found that there are 10 factors with eigenvalues greater than 1, and the first common factor explained 38.549% of the variance, which is below the critical standard of 40%. This indicates no severe homologous bias in this study.

## Research tools

### Physical activity rating scale (PARS-3)

The Physical Activity Rating Scale (PARS-3) was compiled by the Japanese scholar Takao Hashimoto and revised by Liang et al. (46). The scale examines the amount of physical activity, including intensity, frequency, and workout time. It uses them to measure the level of participation in physical activity. The physical activity score = intensity × (time-1) × frequency and each aspect was divided into five levels, scored on a scale of 1 to 5, with a scale of ≤19 points for small exercise, 20–42 points for medium exercise, and ≥ 43 points for extensive training. The PARS-3 scale comprises three dimensions: Intensity, frequency, and duration. Physical activity level = Intensity x Duration x Frequency, with Intensity and frequency graded from 1 to 5, each assigned 1–5 points respectively, and duration graded from 1 to 5, each assigned 0–4 points, respectively. The highest score is 100 points, and the lowest score is 0 points. Physical activity level assessment standards: ≤19 points are classified as fluctuating activity level; 20–42 points as moderate activity level; ≥43 points as high activity level. In the “Exercise Intensity” dimension, the number 1 signifies “Minimal Intensity,” the number 2 “Low Intensity,” the number 3 “Moderate Intensity,” the number 4 “High Intensity,” and the number 5 “Maximum Intensity.” In the “Frequency” dimension, the number 1 represents “Less than once a month,” the number 2 “3 to 5 times a week,” the number 3 “2 to 3 times a month,” the number 4 “Approximately once a day,” and the number 5 “1 to 2 times a week.” In the “Duration” dimension, the number 0 indicates “Less than once a month,” the number 1 “3 to 5 times a week,” the number 2 “2 to 3 times a month,” the number 3 “Approximately once a day,” and the number 4 “1 to 2 times a week.” The results of the PARS-3 represent the amount of physical activity of the subjects, and its retest reliability was 0.82. In previous studies, Javalle and Cheng used the exercise scale measure to measure the physical activity participation of different age groups and the status of physical exercise levels. They verified the scale’s reliability, and its reliability level is high (0.70–0.80) (47).

### Depression anxiety and stress scale (DASS-21)

The Depression-Anxiety-Stress Self-rated Scale in Simplified Chinese (DASS-21), compiled by Lovibond et al. (48), revised by Antony et al. (49), and translated by Yuan, was used for the measurement. The Chinese scale version is reliable and valid for the Chinese adolescent population. The scale consists of 21 items, with three subscales for depression, anxiety, and stress, each containing seven items. Scoring ranges from “0” (not applicable) to “3” (always applicable), with higher scores indicating a more substantial presence of these emotions. In the Likert 4-point scoring system, the number “0” represents “Did not apply to me at all”; “1” represents “Applied to me to some degree or some of the time”; “2” represents “Applied to me to a considerable degree, or a good part of the time”; “3” represents “Applied to me very much, or most of the time.” In the dimensions of depression, anxiety, and stress, higher scores on the survey indicate a more severe level of these negative emotions. Analysis of 543 valid preliminary survey questionnaires revealed that the scale’s Cronbach’s alpha coefficient is 0.891, KMO value is 0.925, and the Cronbach’s alpha coefficients for the depression, anxiety, and stress subscales are 0.774, 0.743, and 0.752 respectively, indicating good reliability and validity of the scale (50, 51).

### Pittsburgh sleep quality index (PSQI)

A revised questionnaire based on the Pittsburgh Sleep Quality Index Scale compiled by Buysse was used to assess the sleep quality of college students Liu (52, 53). The scale covers seven dimensions with a total of 19 measurement entries, and a score of 0–3 was used to assess the scores of the measurement entries. The scale was scored reversely, with higher overall scores representing more severe sleep quality problems in individuals, and the scores were generally in the range of 0–21 points. In past studies, sleep quality questionnaires were commonly used among healthcare workers, high-pressure groups, the older adult, and special sleep disorder groups, and the level of reliability and validity was also high (0.77–0.88), reaching the range defined by social science research (54, 55).

### Self-rated health status (Short Form Health Survey-12, SF-12)

Using a single entry from the Short Form Health Survey-12 (SF-12) (Overall, what do you think your current health status is?) Conduct a Self-rating of your health (55). Participants were asked to rate their perceived health (1 = poor, 2 = fair, 3 = good, 4 = very sound, 5 = very good), categorizing self-rated health scores as ≥3 (good, very good, or excellent) and < 3 (poor or fair). The scale’s internal consistency reliability, Cronbach’s alpha, is 0.84. The correlation coefficients between each dimension and the total score are above 0.50, except for physical functioning (PF) at 0.43. Cronbach’s alpha coefficients for all dimensions exceed 0.70, remaining above 0.70 even after the respective dimensions are removed. The scale’s construct validity was confirmed with a 100% success rate in both convergent and discriminant validity calibration experiments (56). Confirmatory factor analysis of the theoretical structure model yielded a model consistent with original assumptions, with fit indices showing a non-normal fit index (NNFI) of 0.95, a comparative fit index (CFI) of 0.96, an adjusted goodness of fit index (AGFI) of 0.96, and a root mean square error of approximation (RMSEA) of 0.06. Furthermore, the reliability of the SF-12, compared to PCS-12 and MCS-12, was validated, ranging from 0.63 to approximately 0.91 (57).

### Data analysis

#### Data preprocessing in excel

Initially, you use Excel to preprocess the data obtained from Questionnaire Star, addressing missing or problematic data through retesting or deletion.

#### Common method bias test

To prevent issues related to common method bias, you perform tests specifically designed to identify this type of bias, ensuring the validity of your findings.

Analysis of Core Variables with ANOVA and Chi-Square Tests: You conduct a one-way ANOVA and chi-square tests to analyze core variables. The Cramer’s V coefficient, which ranges from 0 to 1, assesses the strength and correlation between two variables. A higher difference suggests a more substantial effect and correlation between the variables.

#### Kendall’s rank correlation analysis

This analysis tests the correlations between physical exercise, sleep quality, self-rated health, stress, anxiety, and depression. Kendall’s W coefficient, ranging from 0 to 1, interprets the degree of correlation between variables based on familiar statistical measures. Values closer to 1 indicate a higher degree of correlation.

The mediation analysis was carried out through model 6 in plug-in process 4.0, and the mediation model was tested with the help of the Bootstrap method for the relevant core variables.

## Results

### Descriptive analysis

Before examining the mechanisms by which physical exercise affects negative emotions in college students, a description of the essential characteristics of each variable was provided, including sample size, percentage, chi-square value, and Cramér’s V coefficient. Results from Table 1 indicate that physical exercise among college students is primarily of low intensity, accounting for 77.6%. Gender-wise, female students have significantly lower physical activity levels than males (*V* = 0.311, *p* < 0.001), with a statistically significant difference. The proportion of low-intensity exercise reached 87.9%, while high-intensity exercise accounted for only 3.3%. Considering the distribution across grades, there is a statistically significant difference in the levels of physical exercise between male and female students across different grades (*V* = 0.021, *p* < 0.001).

**Table 1:** Descriptive statistic variables.

Norm	Assemble	Gender					Grade								
				Male (*n* = 12,440)		Female (*n* = 18,035)		Freshman(*n* = 9,718)		Sophomore		Junior (*n* = 6,406)		Senior (2410)	
										(*n* = 11,941)					
		*n*	*%*	*n*	*%*	*n*	*%*	*n*	*%*	*n*	*%*	*n*	*%*	*n*	*%*
Physical activity level															
	Low	23,643	77.6	7786	62.5	15857	87.9	7518	77.4	9235	77.3	5025	78.4	1865	77.4
	Middle	3986	13.1	2408	19.4	1578	8.8	1351	13.9	1587	13.3	738	11.6	310	12.9
	High	2846	9.3	2246	18.1	600	3.3	849	8.7	1,119	9.4	643	10	235	9.7
	*χ*2			2952.305				25.775							
	*p*			<0.001				<0.001							
	Cramer’s V			0.311				0.021							
Self-rated health															
	Terrible	867	2.8	463	3.7	404	2.2	239	2.5	351	2.9	186	2.9	91	3.8
	After a fashion	11139	36.6	4247	34.1	6892	38.2	3523	36.3	4526	37.9	2225	34.7	865	35.8
	Fine	10070	33.1	3784	30.4	6,286	34.8	3239	33.3	3999	33.5	2047	32	785	32.6
	Excellent	5405	17.7	2424	19.5	2981	16.5	1794	18.4	1993	16.7	1192	18.6	426	17.7
	Super	2994	9.8	1522	12.3	1472	8.3	923	9.5	1072	9	756	11.8	243	10.1
	*χ*2			294.695				76.992							
	*p*			<0.001				<0.001							
	Cramer’s V			0.098				0.029							
Sleep quality															
	Pretty good	13821	45.3	5931	47.7	7890	43.7	4780	49.2	5,258	44.1	2686	41.9	1097	45.5
	General	11,554	37.9	4339	34.9	7215	40	3652	37.6	4550	38.1	2505	39.1	847	35.1
	Poorly	5100	16.8	2170	17.4	2930	16.3	1,286	13.2	2133	17.8	1215	19	466	19.4
	*χ*2			82.387				169.414							
	*p*			<0.001				<0.001							
	Cramer’s V			0.052				0.053							
Pressure rating															
	Normalcy	15888	52.1	5931	47.7	9957	55.2	5566	57.3	5901	49.4	3250	50.7	1171	48.6
	Mildly	8880	29.1	3510	28.2	5370	29.8	2784	28.6	3629	30.4	1778	27.8	689	28.6
	Moderately	4352	14.3	2101	16.9	2251	12.5	1131	11.6	1786	15	1022	15.9	413	17.1
	Severe	1355	4.5			898	7.2	237	2.4	625	5.2	356	5.6	137	5.7
	*χ*2			549.805				294.585							
	*p*			<0.001				<0.001							
	Cramer’s V			0.134				0.057							
Anxiety level															
	Mildly	11024	36.2	4359	35.1	6665	36.9	3547	36.5	4112	34.4	2476	38.7	889	36.9
	Moderately	8491	27.9	2944	23.7	5547	30.8	3264	33.6	3138	26.3	1495	23.3	594	24.6
	Severe	7616	25	3191	25.6	4425	24.5	2247	23.1	3187	26.7	1599	25	583	24.2
	Very Serious	3344	10.9	1946	15.6	1398	7.8	660	6.8	1504	12.6	836	13	344	14.3
	*χ*2			561.824				463.129							
	*p*			<0.001				<0.001							
	Cramer’s V			0.136				0.071							
Depression level															
	Normalcy	6806	22.3	2680	21.6	4126	22.9	2258	23.2	2494	20.9	1491	23.3	563	23.4
	Mildly	9492	31.1	3300	26.5	6192	34.3	3639	37.4	3404	28.5	1814	28.3	635	26.3
	Moderately	10276	33.8	4202	33.8	6074	33.7	3050	31.4	4296	36	2125	33.2	805	33.4
	Severe	2807	9.2	1512	12.2	1,295	7.2	604	6.3	1218	10.2	692	10.8	293	12.2
	Very Serious	1094	3.6	746	5.9	348	1.9	167	1.7	529	4.4	284	4.4	114	4.7
	*χ*2			686.888			525.039								
	*p*			<0.001			<0.001								
	Cramer’s V			0.15			0.076								

Regarding stress levels, there is a statistically significant difference between male and female college students (*η*^2^ = 0.013, *p* < 0.001), with means and standard deviations of 13.51 ± 5.86 and 12.29 ± 4.67, respectively. The correlation in anxiety levels between male and female college students is weak. The difference in depression levels across genders is statistically significant (*η*^2^ = 0.019, *p* < 0.001), with means and standard deviations of 13.03 ± 5.89 and 11.56 ± 4.65, respectively, indicating a higher correlation than for anxiety. The difference in DASS (Depression, Anxiety, and Stress Scale) scores between genders is statistically significant (*η*^2^ = 0.0155, *p* < 0.001), with a low correlation. Evaluating across different grades, first-year students and sophomores exhibit higher levels of depression, with less variability and stable changes (*p* < 0.001), which is statistically significant (Table 2).

**Table 2:** Descriptive statistical analysis.

		Assemble		Gender				Grade							
				Male (*n* = 12,440)		Female (*n* = 18,035)		Freshman (*n* = 9,718)		Sophomore (*n* = 11,941)		Junior(*n* = 6,406)		Senior(2410)	
		*M*	*SD*	*M*	*SD*	*M*	*SD*	*M*	*SD*	*M*	*SD*	*M*	*SD*	*M*	*SD*
Stresses		12.79	5.23	13.51	5.86	12.29	4.67	12.17	4.56	13.08	5.41	13.02	5.57	13.21	5.63
	*F*			408.052				68.57							
	*p*			<0.001				<0.001							
	*η^2^*			0.013				0.007							
Apprehensive		12.39	5.1	13.08	5.77	11.91	4.51	11.82	4.34	12.71	5.31	12.54	5.47	12.7	5.58
	*F*			389.71				60.727							
	*p*			<0.001				<0.001							
	*η^2^*			0.013				0.006							
Despondent		12.16	5.24	13.03	5.89	11.56	4.65	11.29	4.477	12.57	5.443	12.5	5.578	12.75	5.679
	*F*			587.165				135.544							
	*p*			<0.001				<0.001							
	*η^2^*			0.019				0.013							
Total DASS score		37.335	15.21	39.62	17.21	35.76	13.43	35.27	12.9	38.36	15.86	38.06	16.332	38.65	16.57
	*F*			479.984				89.318							
	*p*			<0.001				<0.001							
	*η^2^*			0.016				0.009							

### Correlation analysis

The Kendall rank correlation analysis of the variables, as shown in Table 3, indicates significant negative correlations between physical exercise and stress, anxiety, and depression.

**Table 3:** List of results of correlation analysis.

	Variant		Physical exercise	Stresses	Apprehensive	Despondent	Self-rated health	Sleep quality
Kendall (name)	Physical exercise	*r*	1					
	Stresses	*r*	−0.030**	1				
	Apprehensive	*r*	−0.058**	0.770**	1			
	Despondent	*r*	−0.055**	0.761**	0.774**	1		
	Self-rated health	*r*	0.194**	−0.248**	−0.280**	−0.299**	1	
	Sleep quality	*r*	−0.035**	0.322**	0.341**	0.330**	−0.242**	1

In the text, ** represents *p* < 0.01 and *** represents *p* < 0.001.

### Analysis of mediating effects

The following model was established based on hierarchical regression analysis: physical exercise, self-rated health, and sleep quality as independent variables; gender, grade, smoking, and drinking as control variables; and negative emotion as the dependent variable. The model was designed to test for main, direct, and indirect effects. The results of the hierarchical regression analysis for chained mediation effects are presented in Table 4.

**Table 4:** Hierarchical regression analysis of chained mediation effects.

	Regression equation			Overall fit index		Significance of regression coefficients		
Outcome variable		Predictor variable	*R*	*R^2^*	*F*	*β*	*SE*	*t*
DASS			0.169	0.029	178.672***			
		Gender				−0.132	0.193	−21.128***
		Grade				0.075	0.094	13.160***
		Cigarette smoking				0.055	0.26	9.055***
		Drinking wine				0.01	0.16	1.698
		Physical exercise				−0.071	0.143	−11.894***
DASS			0.477	0.227	1280.918***			
		Gender				−0.139	0.172	−24.915***
		Grade				0.053	0.084	10.340***
		Cigarette smoking				0.026	0.232	4.830***
		Drinking wine				−0.032	0.143	−5.952***
		Physical exercise				−0.018	0.131	−3.251**
		Sleep quality				0.389	0.019	74.068***
		Self-rated health				−0.154	0.079	−28.734***
Sleep quality			0.135	0.018	113.446***			
		Gender				0.014	0.053	2.166*
		Grade				0.063	0.026	11.021***
		Cigarette smoking				0.067	0.071	10.963***
		Drinking wine				0.081	0.044	13.389***
		Physical exercise				−0.044	0.039	−7.306***
Self-rated health			0.337	0.114	650.186***			
		Gender				−0.007	0.0124	−1.13
		Grade				0.03	0.0061	5.446***
		Cigarette smoking				−0.002	0.017	−0.322
		Drinking wine				−0.051	0.01	−8.819***
		Physical exercise				0.225	0.009	39.422***
		Sleep quality				−0.242	0.001	−44.530***

In the text, *** represents *p* < 0.001, ** represents *p* < 0.01, and * represents *p* < 0.05.

The table below shows that controlling for variables such as gender, grade, smoking, and drinking, physical exercise significantly negatively predicts negative emotions, *β* = −0.071, *SE* = 0.143, *t* = −11.894, *p* < 0.001, confirming the main effect. Physical exercise significantly negatively predicts sleep quality, *β* = −0.044, *SE* = 0.039, *t* = −7.306, *p* < 0.001, and significantly positively predicts self-assessed health status, *β* = 0.225, *SE* = 0.009, *t* = 39.422, *p* < 0.001. It also significantly negatively predicts negative emotions, *β* = −0.018, *SE* = 0.131, *t* = −3.251, *p* < 0.01. Self-assessed health significantly negatively predicts negative emotions, *β* = −0.154, *SE* = 0.079, *t* = −28.734, *p* < 0.001; sleep quality significantly positively predicts negative emotions, *β* = 0.389, *SE* = 0.019, *t* = 74.068, *p* < 0.001.

This study utilized a bias-corrected percentile Bootstrap method (with 5,000 Bootstrap samples) and model 6 from Hayes’s (58) *SPSS* macro program, *PROCESS 4.0*. A chained mediation effect analysis was conducted controlling for gender and grade to investigate the mediating roles of self-rated health and sleep quality between physical exercise and negative emotions in college students. The Bootstrap chained mediation effect analysis results are presented in Table 5. The main effect of physical exercise on negative emotions was −1.702, with a 95% confidence interval of [−1.983, −1.422], not crossing zero, indicating that the total effect is significant. The total indirect effect was-1.277, with a 95% confidence interval of [−1.421, −1.128], not crossing zero, accounting for 75.5% of the effect. The total direct effect was −0.426, with a 95% confidence interval of [−0.683, −0.169], not crossing zero, accounting for 24.5% of the effect, thus confirming the direct effect. Specifically, the indirect effect of physical exercise → sleep quality → self-rated health → *DASS* was −0.039, with a 95% confidence interval of [−0.051, −0.027], indicating that the model constitutes a partial chained mediation (Table 5).

**Table 5:** Chain-mediated effects analysis of stress, anxiety, depression, and DASS by Bootstrap method.

Effect (scientific phenomenon)	Efficiency value	BootSE	95% CIlower limit	95% CI upper limit	Proportion of effect
Aggregate effect	−1.702	0.143	−1.983	−1.422	
Total direct effect	−0.426	0.131	−0.683	−0.169	24.50%
Total indirect effect	−1.277	0.075	−1.421	−1.128	75.50%
Physical activity → sleep quality → DASS	−0.408	0.06	−0.523	−0.287	55.40%
Physical activity → Self-rated health → DASS	−0.829	0.04	−0.907	−0.751	8.03%
Physical activity → Sleep quality → Self-rated health → DASS	−0.039	0.006	−0.051	−0.027	28.05%

## Discussion

**Figure 1:**
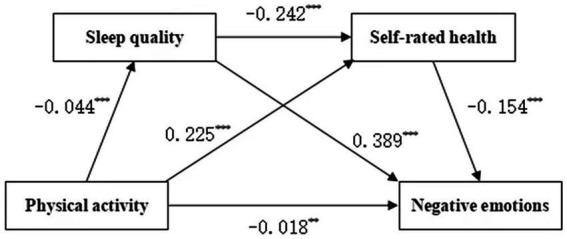
Shows the chain-mediated route coefficients of unpleasant emotions.

This study examined the roles of sleep quality and self-rated health in the effects of physical exercise on negative emotions of Chinese college students and the relationship between physical exercise and negative emotions of college students, which supports hypotheses 1, 2, and 3. Furthermore, this study reveals that college student has potential benefits in enhancing emotional health through active participation in physical activity. Physical exercise can affect negative emotions through the chain mediating effects of sleep quality and self-rated health, which means that the effects of physical exercise on college student’s mental health are interfered with by the chain mediating roles of sleep and self-cognition, which provides a scientific basis for the design of intervention programs for college students’ mental health (Figure 1).

### Correlation of physical activity, sleep quality, self-rated health, and negative emotions among college students

This study found that physical exercise is negatively correlated with college students’ stress, anxiety, and depression, negatively correlated with sleep quality, and positively correlated with self-rated health. After incorporating mediating variables, the predictive role of physical exercise on negative emotions in college students remained significant, thus confirming hypothesis *H1*. Academic activities occupy a crucial position in the lives of college students. At the same time, participation in physical exercise offers opportunities to enhance individual self-esteem levels, subjective well-being, and interpersonal skills (59). Sleep is fundamental for college students to maintain normal physiological functions and social activities. Poor sleep habits, such as staying up late, frequent napping, and excessive reliance on sleeping pills, can affect their daily life, learning, and peer relationships (60). Self-rated health is based on individuals’ understanding of their physiological, psychological, and social adaptability, integrating subjective and objective health information to form an overall perception of their health status (30, 61, 62). The results of this study show a negative correlation between sleep quality and self-rated health. An influential relationship exists between sleep duration and brain cognitive function, emotional activities, and the duration of social interactions (63). However, support from longitudinal data or follow-up survey data is needed to verify these causal relationships. Internationally, based on Brown’s theory, research on sleep quality and self-rated health often focuses on responses to different stresses, handling emergencies, and preventing aggressive behaviors (64). Rarely do studies treat sleep quality and self-rated health among youth as continuous variables to examine their relationship with negative emotions. Therefore, physical exercise can promote the development of mental health in college students and prevent the occurrence of negative emotions.

### The direct effect of sports on negative emotions of Chinese college students

The findings of this study demonstrate that physical activity can significantly positively affect college students’ negative emotions. The neuroprogression hypothesis suggests that long-term, regular physical exercise can significantly counteract the progression of mood disorders by enhancing the expression of neurotrophic factors in the brain and reducing stress-induced neuroinflammatory responses (65). The key motivation for college students to persist in physical exercise is self-efficacy and self-esteem. Gaining a sense of achievement and belief can help reduce psychological stress among college students. According to Rosenberg’s self-esteem theory, individual participation in exercise, achievement of exercise goals, and completion of corresponding challenges can provide a psychological buffer and enhance positive emotional experiences through perceived self-worth (66). According to Ulrich’s Stress Recovery Theory, when college students engage in outdoor physical exercises, the natural environment can help replenish cognitive resources depleted by prolonged focus (67). Once the duration and intensity of physical exercise reach a certain level, individuals can alleviate fatigue, restore attention, and enhance their emotional regulation abilities in the natural setting (68).

### Mediating effects of sleep quality

The results of this study indicate that sleep quality mediates the relationship between physical exercise and negative emotions among college students. Physical activity can directly influence students’ negative emotions and indirectly affect their psychological health through good sleep quality, thus confirming research hypothesis *H2*. Siegel’s cross-cultural study suggests that the function of sleep may be to enhance behavioral efficiency during periods when biological activity is no longer beneficial by optimizing time use and reducing energy consumption (69). Under neural regulation, improving individual perception and physiological functions helps mitigate the adverse effects on the body caused by sleep-disordered breathing and circadian rhythm disorders (70). According to circadian rhythm theory, college students who achieve higher sleep quality through physical exercise have an enhanced ability to monitor and accept current psychological experiences, enabling them to cope with negative emotional experiences triggered by negative stimuli more quickly (71). Alexander Borbély’s Two-Process Model of sleep regulation corroborates the two main processes of sleep modulation, sleep propensity, and circadian rhythms, laying a solid foundation for enhancing mental health quality (72). Shang’s research indicates that engaging in aerobic exercise with peers reduces sensitivity and bias in interpersonal relationships (73). Improving interpersonal relations can help alleviate evening anxiety and stress, enhance sleep quality, and reduce the physical and mental fatigue caused by stress and anxiety in the college student population.

### The mediating role of self-rated health

The results of this study show a strong correlation between self-rated health and physical exercise. The path analysis between self-rated health and negative emotions reveals a significant effect of self-rated health. Studies have shown that the overall self-rated health score is positively correlated with the total health literacy score; the higher the self-rated health score, the higher the individual’s level of health literacy (74). Individuals with high health literacy are more likely to adopt positive health behaviors and coping strategies when facing mental health issues. There are statistically significant differences in the frequency of participation in physical exercise, self-rated health status, lifestyle, and behavioral literacy levels among college students of different ages, genders, and grades show statistically significant differences (75, 76). Strengthening education on healthy lifestyles, behaviors, and primary health skills among college students can effectively enhance their awareness and ability to prevent infectious and chronic diseases. Other studies analyze individuals’ subjective perceptions of their health status from the perspectives of intergenerational relationships and social support, where emotional resonance from relatives and financial assistance from the government or society partially mediate between self-rated health and anxiety (77, 78). This study examines the understanding of health definitions among college students and the importance of self-rated of health status. Forming health awareness and good health concepts among college students can help cultivate regular rest, a reasonable diet, and moderate exercise habits.

### Analysis of the chain mediating role of sleep quality and self-rated health in physical activity on negative emotions among college students

This study constructed a mediation model for the impact of physical exercise on negative emotions among college students, with sleep quality and self-rated health as mediating variables. According to the test of chained mediation effects, physical exercise can not only directly negatively predict college students’ negative emotions but can also indirectly influence negative emotions through the mediating roles of sleep quality and self-rated health, thus confirming hypothesis *H3*. The symptoms of depression and anxiety represented by negative emotions partially explain the association between sleep quality and self-rated health. This association operates to some extent through an increase in the levels of depression and anxiety symptoms (79). According to the resource depletion theory, student groups under significant daily stress experience greater consumption of emotional and physical energy resources, leading to varying degrees of impact on their self-control abilities (80, 81). The emergence of stress results from college students being exposed to excessive external stimuli over time, which elevates their sensitivity to adverse life events and diminishes their capacity for emotional expression and control. Given the intrinsic link between physical exercise and anxiety, short-term moderate to high-intensity physical exercise can produce instantaneous emotional improvement (82). This efficient, emotional enhancement positively affects sleep quality. Ensuring adequate sleep duration significantly influences college students’ perception of their health (14, 83, 84).

However, a meta-analysis indicated that sleep duration—whether equal to, exceeding, or less than 8 h—impacts self-rated health to various extents and is associated with increased rates of fatigue and depression (85). Upon entering college, students’ independence and psychological resilience are challenged, leading to an unavoidable decrease in subjective well-being due to the social environment. Short-term experiences of “flow” can produce feelings of pleasure and self-efficacy, alleviating anxiety and stress (86, 87). The dense college life, filled with opportunities and challenges, can lead to a decline in mood when students do not achieve good results in exams and other competitions. The downturn in emotions suppresses the release of monoamine neurotransmitters (88). Engaging in sports can effectively divert the attention of the college student group from adverse events, producing the pleasure of exercise.

Spiritual abundance can enhance the college student group’s satisfaction with life and reduce the occurrence of negative emotions (89). According to Symbolic Interaction Theory, an individual’s self-cognition is primarily constructed through interactions with others (90). Frequent and effective interactions can satisfy an individual’s psychological needs, improving self-rated health (91). To properly handle the negative impact of negative emotions, college students need to maintain long-term exercise compliance and sufficient sleep, manage themselves appropriately and promptly, and reduce levels of physical stress hormones. Only in this way can a lasting impact on college students’ mental and physical health be achieved.

### Research limitations

The study’s limitations are that self-reporting can obtain participants’ subjective feelings and opinions and can quickly and easily convey the study results. Still, there may be response bias in the reported data, and the individual’s cognitive level and emotional state may affect the results to a certain extent. In addition, cross-sectional studies do not yield exact causal relationships, so researchers need to expand the sample size and incorporate current big data technology to design better interventions for negative emotions among college students and improve the efficiency of the study.

## Conclusion

This study aims to understand whether engaging in physical activities can improve emotional states by enhancing sleep and the overall health perception of students, thereby reducing their experience of negative emotions such as stress, anxiety, and depression. Physical exercise has a positive impact on negative emotions in university students. Moreover, sleep quality and self-rated health play a chain mediating role in the effect of physical exercise on these negative emotions. This study extends existing research on the relationship between physical exercise and mental health by exploring the interactions among physical exercise, sleep quality, self-rated health, and negative emotions, providing new insights into how physical exercise impacts emotions through multiple pathways.

The study introduces a chain mediation model to demonstrate how sleep quality and self-rated health act as mediating variables in the influence of physical exercise on negative emotions in university students, enriching the theoretical framework of the psychological effects of physical exercise and providing new hypotheses for future research. Additionally, this study encourages higher education institutions and policymakers to increase their focus on university students’ lifestyles, behavioral habits, and stress resilience and to consider the potential moderating factors in the bidirectional interaction between physical activity and mental health when designing personalized emotional intervention plans.

## Data availability statement

The data analyzed in this study is subject to the following licenses/restrictions: Since this data contains some privacy related to college students’ mental health issues, it can be obtained from the corresponding author if necessary. Requests to access these datasets should be directed to wangqiulibo@163.com.

## Ethics statement

The studies involving humans were approved by General Program of Education of the National Social Science Fund of China: “Research on sports regulation mechanism and intervention scheme of middle school students’ psychological pressure.” The studies were conducted in accordance with the local legislation and institutional requirements. The participants provided their written informed consent to participate in this study.

## Author contributions

F-zM: Data curation, Formal analysis, Investigation, Methodology, Writing – original draft, Writing – review & editing. JL: Methodology, Resources, Writing – review & editing. HL: Funding acquisition, Resources, Writing – review & editing. W-dZ: Data curation, Validation, Writing – review & editing. Z-cW: Methodology, Writing – review & editing. BL: Data curation, Funding acquisition, Methodology, Resources, Writing – review & editing.
